# Efficacy and safety of calcineurin inhibitors (CNIs) for septic patients in ICU: a cohort study from MIMIC database

**DOI:** 10.3389/fphar.2024.1394553

**Published:** 2024-09-18

**Authors:** ShengHui Miao, Mingkun Yang, Wen Li, Zhouxin Yang, Jing Yan

**Affiliations:** ^1^ The Fourth Affiliated Hospital, International Institutes of Medicine, Zhejiang University School of Medicine, Yiwu, China; ^2^ Department of Second Clinical Medical College, Zhejiang Chinese Medicine University, Hangzhou, Zhejiang, China; ^3^ Department of Critical Care Medicine, Zhejiang Hospital, Zhejiang University School of Medicine, Hangzhou, Zhejiang, China

**Keywords:** sepsis, calcineurin inhibitors (CNIs), 28-day mortality, cohort study, adverse reaction

## Abstract

**Background:**

Sepsis is marked by a dysregulated immune response to infection. Calcineurin inhibitors (CNIs), commonly used as immunosuppressants, have unique properties that may help mitigate the overactive immune response in sepsis, potentially leading to better patient outcomes. This study aims to assess whether CNIs improve prognosis in septic patients and to evaluate any associated adverse reactions.

**Methods:**

We utilized the Medical Information Mart for Intensive Care IV 2.2 (MIMIC-IV 2.2) database to identify septic patients who were treated with CNIs and those who were not. Propensity score matching (PSM) was employed to balance baseline characteristics between the CNI user group and the non-user group. The primary outcome was 28-day mortality, analyzed using the Kaplan-Meier method and Cox proportional hazard regression models to examine the relationship between CNI use and patient survival.

**Results:**

From the MIMIC-IV database, 22,517 septic patients were identified. After propensity score matching, a sample of 874 patients was analyzed. The CNI group exhibited a significantly lower 28-day mortality risk compared to the non-user group (HR: 0.26; 95% CI: 0.17, 0.41) in the univariate Cox hazard analysis. Kaplan-Meier survival curves also demonstrated a significantly higher 28- and 365-day survival rate for CNI users compared to non-users (log-rank test p-value = 0.001). No significant association was found between CNI use and an increased risk of new-onset infection (p = 0.144), but an association with mild hypertension (P < 0.001) and liver injury (P < 0.001) was observed.

**Conclusion:**

The use of calcineurin inhibitors was associated with reduced short- and long-term mortality in septic patients without an increased incidence of new-onset infections, hyperkalemia, severe hypertension, or acute kidney injury (AKI). However, CNI use may lead to adverse effects, such as liver injury and mild hypertension.

## 1 Introduction

Sepsis is currently defined as life-threatening organ dysfunction resulting from a dysregulated host response to infection (Singer et al., 2016; Evans et al., 2021). A study published in *The Lancet* estimated that from 1990 to 2017, there were approximately 48.9 million cases of sepsis worldwide, with 11 million resulting in death. This accounts for 19.7% of global mortality (Rudd et al., 2020), highlighting the significant threat sepsis poses to human health and the substantial economic burden it imposes (Maurizio et al., 2018). The dysregulated immune-inflammatory response is central to the development of organ dysfunction and poor outcomes in sepsis. This has made immune modulation therapy a key area of research in recent years (van der Poll et al., 2017). Numerous immunomodulatory agents are currently used as adjunctive treatments for sepsis, ranging from conventional glucocorticoids to various immune checkpoint inhibitors (Sprunge et al., 2008; Djillali et al., 2018; Fabienne and Guillaume, 2018; Marik, 2018). Calcineurin inhibitors (CNIs), which include cyclosporine A (CsA) and tacrolimus (FK506), are widely used immunosuppressants in clinical practice, particularly following organ transplantation and in the treatment of anaphylaxis and autoimmune diseases (Lane and Gbadegesin, 2023; O Grady et al., 2002; Rafael-Vidal et al., 2021). Calcineurin, a phosphatase enzyme, is crucial in immune activation. By dephosphorylating Nuclear factor of activated T-cells (NFAT) family transcription factors, it promotes T-cell differentiation and activation, stimulates the production of inflammatory factors, recruits inflammatory cells to affected tissues, and provides co-stimulation to B cells (Crabtree and Schreiber, 2009; Chen et al., 2022). Given these mechanisms, CNIs have the potential to mitigate the excessive immune-inflammatory response associated with sepsis, potentially reducing organ damage and improving patient outcomes. However, CNIs may also increase the risk of infection, lead to new infections, and cause serious adverse effects, such as liver and kidney damage (Ahmed et al., 2021; Emal et al., 2019; Deppermann et al., 2021). Therefore, it is essential to assess the safety and efficacy of CNIs in septic patients, yet research in this area is currently limited. To address this gap, we aim to conduct an observational cohort study using the Medical Information Mart for Intensive Care IV 2.2 (MIMIC-IV 2.2) database. Our goal is to provide more specific recommendations for the clinical use of CNIs in septic patients and to evaluate the overall benefits and potential risks associated with their use.

## 2 Methods

### 2.1 Data source

The data for this study were obtained from the Medical Information Mart for Intensive Care IV (MIMIC-IV v2.2) database (https://physionet.org/content/mimiciv/2.2/), a freely accessible critical care database comprising 730,141 ICU admissions between 2008 and 2019 at Beth Israel Deaconess Medical Center, United States. The database contains demographics, laboratory measurements, medications, survival data and more. Use of the database was approved by the Institutional Review Boards of MIT and Beth Israel Deaconess Medical Center. As the database is anonymized and contains standardized data, this study did not require separate ethics approval per the Declaration of Helsinki (Johnson et al., 2023a; Johnson et al., 2023b).

### 2.2 Study population

Patients diagnosed with sepsis based on Sepsis-3 criteria were included. Exclusion criteria were: 1) ICU length of stay <24 h due to discharge or death; 2) Age <18 years; and 3) Diagnosis of acquired immunodeficiency syndrome (AIDS). For patients with multiple ICU admissions, only the first admission was analyzed.

Patients who received administration of calcineurin inhibitors (CNIs), including cyclosporin A (CsA) or tacrolimus (FK506), during their ICU stay were defined as the CNIs group. The remaining patients who did not receive CNIs were defined as the control group.

### 2.3 Variables and outcome

We extracted several variables from the MIMIC-IV database for analysis, including demographic information (age, gender, race), clinical measurements on admission (body weight, mean arterial pressure (MAP), white blood cell count (WBC)), illness severity scores (SOFA, SAPSII), comorbidities (chronic heart failure (CHF), cancer, chronic obstructive pulmonary disease (COPD), rheumatism, chronic kidney disease (CKD)), and treatments received (vasopressor drugs, invasive ventilation, continuous renal replacement therapy (CRRT), glucocorticoids).

The primary endpoint was 28-day mortality. Secondary outcomes included utilization of treatments, length of ICU and hospital stay, other mortality timepoint, and safety outcomes. Treatment measures assessed included use of invasive ventilation, vasopressor drugs, and CRRT. Mortality outcomes examined were 28-day, 90-day, 180-day, 1-year, and in-hospital mortality. Safety outcomes were assessed and encompassed new infections during the ICU stay, acute kidney injury (AKI), liver injury, hypertension, and hyperkalemia. AKI was defined in accordance with the KDIGO criteria (Ostermann et al., 2020; Levey et al., 2020), involving an increase in serum creatinine of ≥0.3 mg/dL (≥26.5 μmol/L) within 7 days compared to baseline, or a ≥1.5 times increase in serum creatinine within the same timeframe, or a urine output ≤0.5 mL/kg/h for 6 h. Liver injury was characterized by serum alanine aminotransferase (ALT) or aspartate aminotransferase (AST) levels exceeding 5 times the upper limit of normal. Hypertension was identified as systolic blood pressure exceeding 140 mmHg or diastolic blood pressure exceeding 90 mmHg. Severe hypertension was specifically defined as systolic blood pressure over 180 mmHg or diastolic blood pressure over 110 mmHg. Hyperkalemia was defined as a serum potassium level surpassing 5.5 mmol/L.

### 2.4 Statistical analysis

For continuous variables with non-normal distribution, medians and interquartile ranges were reported, and the Mann-Whitney U test was used for comparisons. Categorical variables were presented as counts and proportions, with chi-square tests used to compare the two groups.

A Cox regression model was constructed in the original cohort to explore the relationship between CNI use and 28-day mortality, with all baseline variables included for multivariate adjustment.

Propensity score matching (PSM) was utilized to control for confounding between the groups. A generalized linear model determined propensity scores for each patient based on selected covariates (age, sex, weight, illness severity scores, MAP, WBC, oxygen saturation, treatments, comorbidities). Patients were matched at a 1:2 ratio using nearest neighbor matching with a 0.2 caliper. Standardized mean differences (SMDs) were calculated between the matched groups.

Kaplan-Meier curves displayed 28-day and 365-day mortality incidence and median survival times in the CNIs-use and control groups. The Cox proportional-hazards model was used to estimate the relationships between patients treated or untreated with CNIs and the 28-day mortality, hazard ratios (HR) and 95% confidence intervals (CI) were analyzed. For subgroup analyses, the study population was stratified based on age, sex, race, WBC levels, SOFA score, CHF, COPD, CKD, organ transplantation status, invasive ventilation use, vasopressor drugs, and glucocorticoid use.

## 3 Results

### 3.1 Baseline characteristics

**FIGURE 1:**
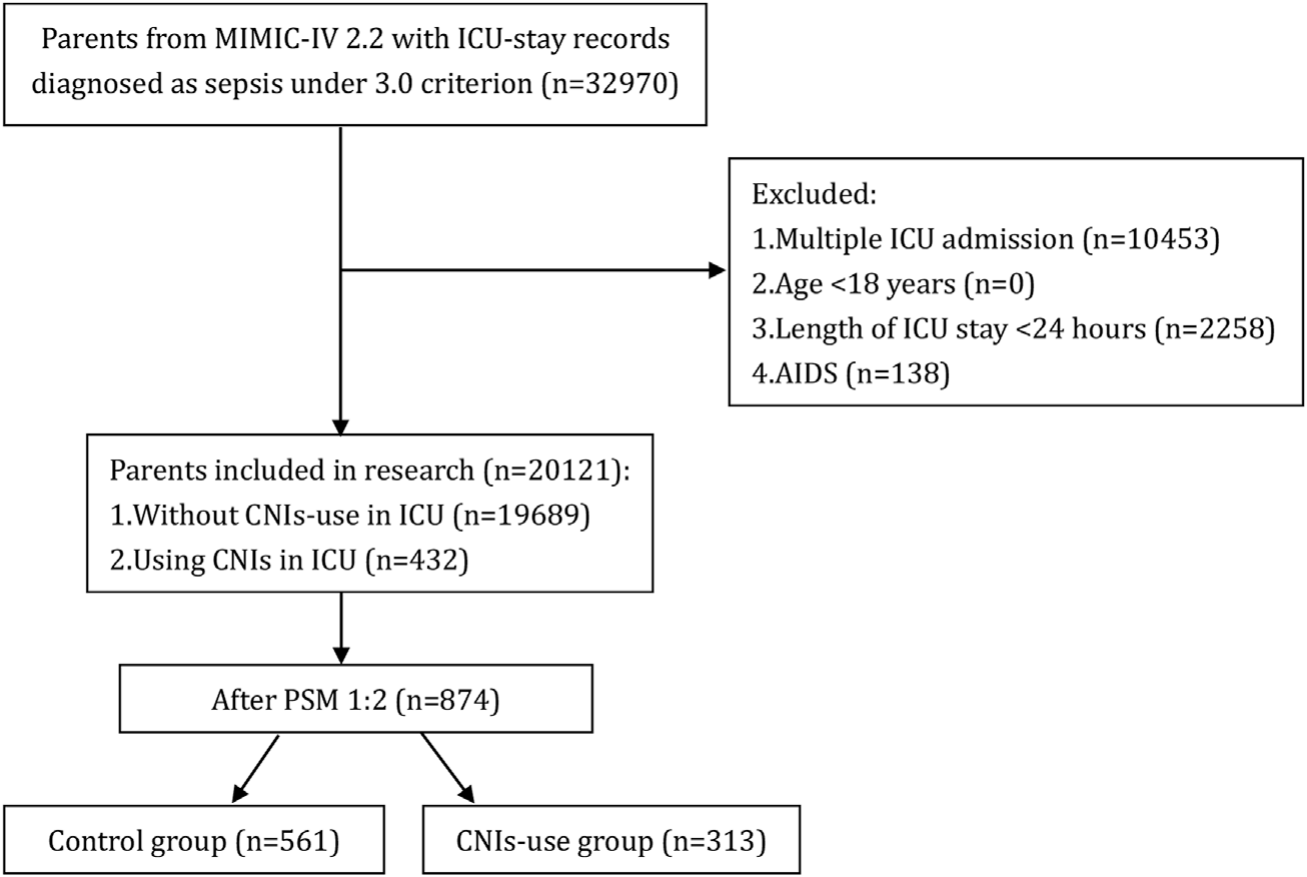
Flow chart of patient selection. MIMIC-IV, Medical Information Mart for Intensive Care IV; AIDS, acquired immune deficiency syndrome; CNIs, Calcineurin inhibitors; ICU, intensive care unit.

A total of 22,517 patients with sepsis admitted to the ICU were screened from the MIMIC-IV database. Based on the predefined inclusion and exclusion criteria, 20,121 patients were included in the final analysis, with 432 patients receiving calcineurin inhibitors (CNIs) and 19,689 not receiving CNIs (Figure1).

Compared to the control group, the CNIs-use group had a higher proportion of males, higher mean arterial pressure, oxygen saturation, comorbidity index, SAPS II and SOFA scores, and greater use of CRRT. The CNIs-use group also had a higher prevalence of kidney disease, malignant tumors, and organ transplantation, while having a lower mean age, white blood cell count, and prevalence of heart failure and COPD (all *p* < 0.05). After 1:2 propensity score matching, the matched cohort consisted of 874 patients, including 313 who received CNIs and 561 who did not. Standardized mean differences for all observed variables were less than 0.1, indicating good balance between the two matched groups. Table 1 shows these baseline characteristics.

**TABLE 1:** Comparisons of baseline characteristics between the original cohort and matched cohort.

Variables	Before matching			After matching		
	Control	CNIs group	SMD△	Control	CNIs group	SMD△
N	19,689	432		561	313	
Age (Year)	66.65 (16.30)	58.94 (11.60)	−0.665	57.40 (16.52)	58.39 (11.38)	0.092
Gender (Male)	11,382 (57.8)	289 (66.9)	0.193	398 (70.9)	211 (67.4)	−0.044
Weight (Kg)	83.27 (23.83)	84.12 (21.64)	0.039	86.02 (23.80)	84.60 (22.14)	−0.010
MAP (mmHg)	76.61 (9.99)	81.73 (11.79)	0.434	81.44 (13.09)	82.00 (12.11)	0.033
SpO2(%)	97.09 (2.20)	97.47 (1.86)	0.209	97.62 (1.86)	97.56 (1.87)	−0.026
WBC(*10 ^ 9/L)	15.84 (12.32)	12.94 (7.13)	−0.407	13.07 (7.40)	13.26 (7.56)	0.031
Hyper-WBC	14,896 (75.7)	263 (60.9)	−0.303	339 (60.4)	193 (61.7)	0.039
SOFA	5.84 (3.45)	7.79 (3.59)	0.544	8.22 (4.15)	8.04 (3.70)	−0.031
SAPSII	39.95 (14.02)	41.55 (13.47)	0.119	40.80 (15.52)	41.55 (13.63)	0.057
CCI	5.04 (2.94)	5.69 (2.41)	0.268	5.52 (3.31)	5.55 (2.29)	0.014
CHF (%)	5,137 (26.1)	72 (16.7)	−0.253	91 (16.2)	50 (16.0)	−0.013
COPD (%)	5,657 (28.7)	88 (20.4)	−0.208	85 (15.2)	57 (18.2)	0.024
Cancer (%)	2,555 (13.0)	123 (28.5)	0.343	188 (33.5)	104 (33.2)	0.011
Rheumatism (%)	705 (3.6)	12 (2.8)	−0.049	15 (2.7)	9 (2.9)	−0.019
CKD (%)	3,986 (20.2)	220 (50.9)	0.614	243 (43.3)	136 (43.5)	−0.048
Transplantation (%)	59 (0.3)	189 (43.8)	0.876	58 (10.3)	71 (22.7)	0.084
Vasopressor (%)	1,312 (6.7)	26 (6.0)	−0.027	24 (4.3)	20 (6.4)	0.074
Invasive MV (%)	11,333 (57.6)	257 (59.5)	0.039	373 (66.5)	200 (63.9)	−0.003
CRRT (%)	559 (2.8)	53 (12.3)	0.287	53 (9.4)	40 (12.8)	0.073
GC-use (%)	1854 (9.4)	52 (12.0)	0.081	79 (14.1)	39 (12.5)	−0.069

SMD, standardized mean difference; MAP, mean arterial pressure; WBC, write blood cell; SOFA, sequential organ failure assessment; SAPSII, simplified acute physiology score; CCI, charlson comorbidity index; COPD, chronic obstructive pulmonary disease; CKD, chronic kidney disease; MV, mechanic ventilation; CRRT, continuous renal replacement therapy; GC, glucocorticoid.

### 3.2 Primary outcomes

**FIGURE 2:**
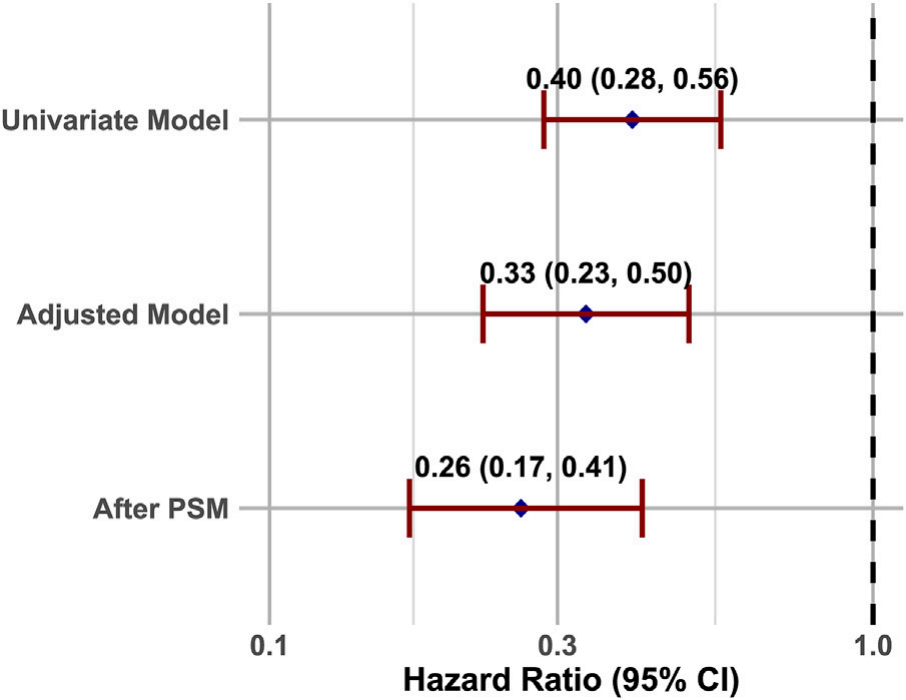
COX analysis of the relationship between CNIs-use and 28-Day mortality, illustrated by a forest plot.

**FIGURE 3:**
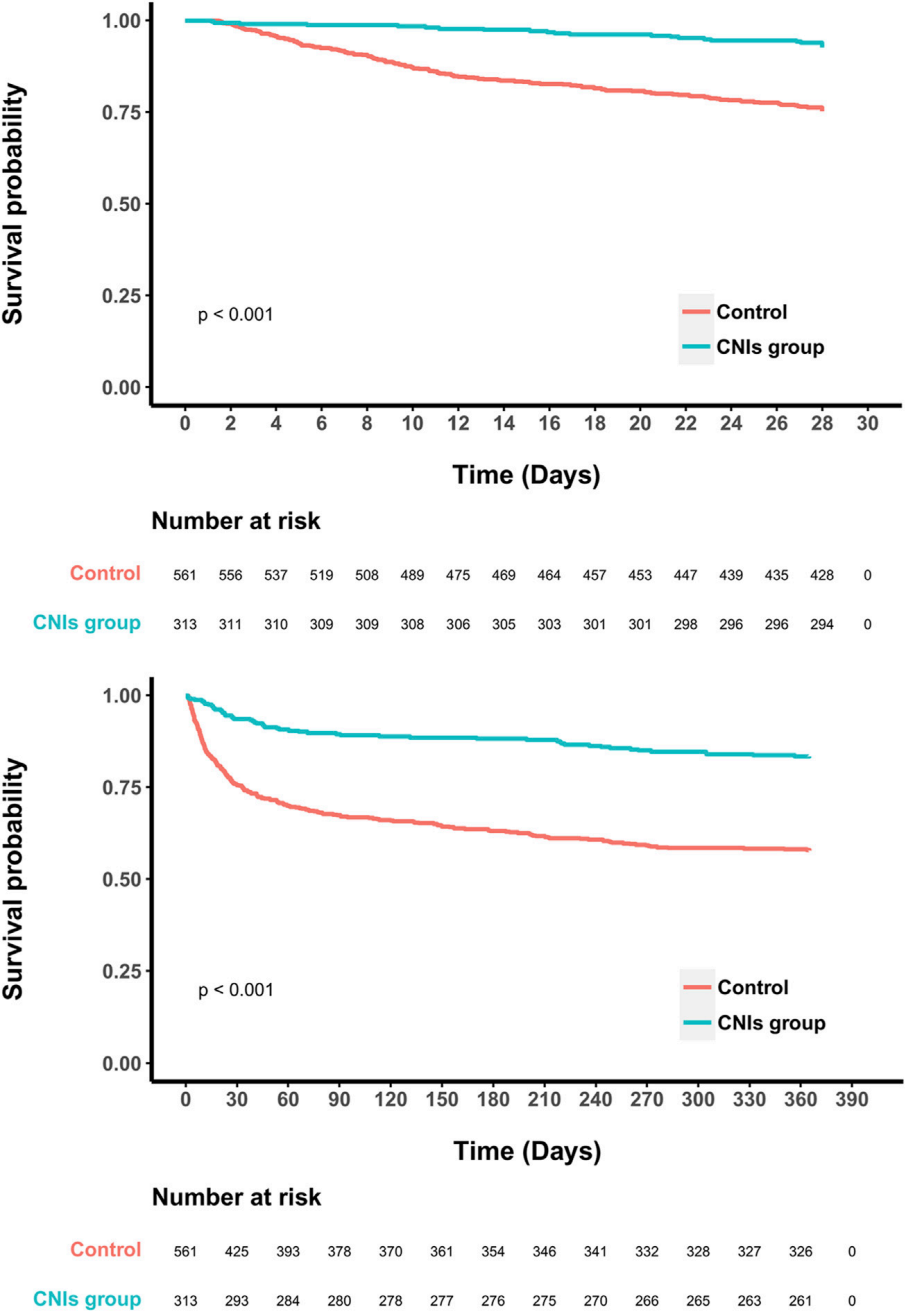
Kaplan-Meier survival curves between two groups indicated the 28- and 365- day mortality risk for the septic patients.

In the original cohort, the use of CNIs was associated with a reduced 28-day mortality rate (HR = 0.40, 95% CI = 0.28–0.56; *p* < 0.001). This association remained significant after including all baseline variables in a multivariate regression model (HR = 0.33, 95% CI = 0.23–0.50; *p* < 0.001) (Figure 2). In the propensity score matched cohort, the 28-day all-cause mortality was 7.3% (23/313) in the CNIs-use group compared to 24.8% (139/561) in the control group (Table 2). Kaplan-Meier survival curves were generated using the log-rank test to evaluate the effect of CNIs treatment (Figure 3). The CNIs group had significantly higher cumulative survival rates compared to the control group (*p* < 0.01). In Cox proportional hazards regression models, CNIs-use group was associated with lower 28-day all-cause mortality (HR = 0.26, 95% CI = 0.17–0.41; *p* < 0.001). (Figure 2).

**TABLE 2:** Association of CNIs use and outcomes in septic patients.

Outcome	Control	CNIs group	*p*
n	561	313	
Mortality			
28-day (%)	139 (25%)	23 (7.3%)	<0.001
90-day (%)	183 (33%)	34 (11%)	<0.001
180-day (%)	208 (37%)	37 (12%)	<0.001
1-year (%)	237 (42%)	52 (17%)	<0.001
Hospital (%)	114 (20%)	23 (7.3%)	<0.001
Life-support treatment			
24-h weaned from Invasive MV (%)	290 (52%)	193 (62%)	0.006
3-day weaned from Invasive MV (%)	402 (72%)	250 (80%)	0.009
Invasive MV-free days (Median (IQR))	27 (17, 28)	27 (25, 28)	0.002
6-h weaned from vasopressors (%)	499 (89%)	293 (94%)	0.032
24-h weaned from vasopressors (%)	518 (92%)	300 (96%)	0.059
Vasopressor-free days (Median (IQR))	28 (28, 28)	28 (28, 28)	0.118
CRRT (%)	69 (12%)	52 (17%)	0.095
Adverse outcome			
New onset of infection (%)	164 (29%)	76 (24%)	0.135
AKI (%)	462 (82%)	257 (82%)	>0.999
Hypertension (%)	421 (76%)	227 (90%)	<0.001
Severe hypertension (%)	191 (34%)	123 (40%)	0.114
Liver injury	98 (31%)	121 (52%)	<0.001
Hyperkalemia	74 (15%)	53 (18%)	0.281
Potassium (mean (SD))	4.07 (0.51)	4.26 (0.47)	<0.001
Length of stay			
ICU (Median (IQR))	3.7 (2.1, 7.9)	3.1 (1.9, 6.9)	0.111
Hospital (Median (IQR))	10 (6, 19)	12 (7, 21)	0.001

CNIs, calcineurin inhibitors; ICU, intensive care unit; MV, mechanical ventilation; AKI, acute kidney injury; CRRT, continuous renal replacement therapy.

### 3.3 Secondary outcomes

Compared to the control group, the CNIs-use group had lower 90-day mortality, 180-day mortality, in-hospital mortality, and longer hospital stays. For mechanical ventilation outcomes, a higher proportion of the CNIs-use group were extubated within 24 h and 3 days, and they had more ventilator-free days versus the control group. Regarding vasopressor use, a higher proportion of the CNIs-use group were weaned off vasopressors within 6 h compared to controls. There was no significant difference between the two groups in utilization of renal replacement therapy.

In terms of adverse effects, the CNIs-use group had a higher incidence of hypertension and liver injury, and a mild increasing of serum potassium compared to the control group. However, there were no significant differences between the groups in new onset of infection, severe hypertension, AKI, or hyperkalemia (Table 2).

### 3.4 Subgroup analyses

**FIGURE 4:**
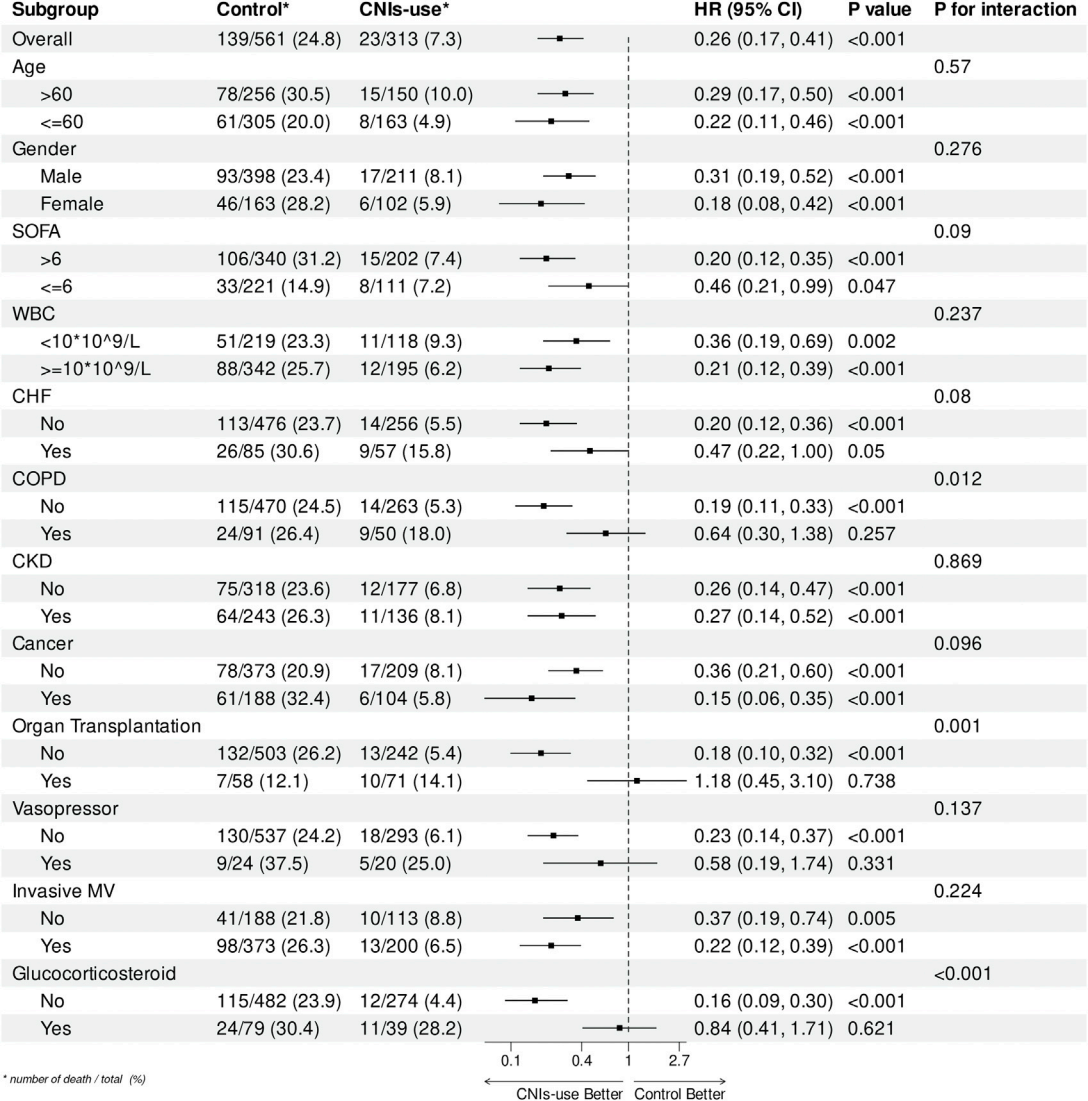
Subgroup analysis of the relationship between CNIs-use and 28-Day mortality, illustrated by a forest plot.

Figure 4 displays the results of a subgroup analysis of 28-day all-cause mortality in the propensity score matched cohort. The association between CNIs use and lower 28-day mortality was consistent across most subgroups, with significant interactions observed only in patients with COPD, organ transplantation, and glucocorticoid use. Specifically, CNIs use was associated with reduced mortality in patients without COPD (HR = 0.19, 95% CI = 0.11–0.33; *p* = 0.012), without glucocorticoid use (HR = 0.16,95% CI = 0.09–0.30; *p* < 0.001), and without organ transplantation (HR = 0.18, 95% CI = 0.10–0.32; *p* < 0.001).

## 4 Discussion

Our study demonstrates that the use of CNIs in septic patients is associated with lower 28- and 365-day mortality, earlier weaning from invasive mechanical ventilation and vasopressors, and only mild side effects. Importantly, there was no significant association with new infections or other severe adverse reactions. Additionally, the use of CNIs enables patients to be liberated from mechanical ventilation earlier, suggesting a better overall condition and reduced dependence on organ function support (Jaber and De Jong, 2023). The partial interactions observed in the subgroup analysis could be attributed to the smaller sample sizes within these subgroups.

This marks the first clinical cohort study to investigate the use of CNIs in septic patients. Although CNIs are extensively used to prevent organ transplant rejection and treat various immune-related diseases (Lane and Gbadegesin, 2023; O Grady et al., 2002; Rafael-Vidal et al., 2021), research on their use in sepsis is scarce. While the role of immune dysregulation and inflammation in sepsis progression has been long recognized, research into anti-inflammatory and immunomodulatory treatments remains a recent focus, and studies specifically examining CNIs as an immunosuppressive option in sepsis are limited. The anti-inflammatory and immunomodulatory effects of CNIs are extensive. For instance, cyclosporine A (CsA) exerts its effects by binding to cyclophilin A (Cyp-A), thereby preventing the nuclear translocation of activated nuclear factor of activated T-cells (NF-AT). This action reduces the transcription of pro-inflammatory cytokines such as interleukin-2 (IL-2), thereby mitigating the inflammatory response during infection. Additionally, when CsA binds to cyclophilin D (CypD), it prevents the opening of the mitochondrial permeability transition pore (mPTP), a critical step in cell death. This protective effect helps reduce cellular dysfunction and death (as shown in the right panel) (Liddicoat and Lavelle, 2019; Cour et al., 2020). Studies have found that CsA’s protective effect on lung injury is dependent on inhibiting 1-methyl-4-phenyl-1,2,3,6-tetrahydropyridine (MPTP) and the release of cytochrome C (CytC), thereby suppressing the activation of the mitochondrial apoptosis pathway and the expression of apoptosis-related proteins, as well as reducing the expression levels of Adenine nucleotide translocator 1 (ANT1) and voltage-dependent anion channel 1 (VDAC1) (Fonai et al., 2015; Li et al., 2017). Previous research has demonstrated that non-cytotoxic concentrations of cyclosporine can inhibit the replication of various viruses, including coronaviruses, hepatitis viruses, and HIV (Natacha et al., 2022; Wang et al., 2014). During the COVID-19 pandemic, cyclosporine significantly reduced key serum cytokines and chemokines in cases of excessive inflammation, leading to lower mortality rates in severe COVID-19 patients (Cour et al., 2020; Galvez-Romero et al., 2021; Rudnicka et al., 2020; Emily et al., 2022).

For septic patients in the ICU, the primary concern with using CNIs is the potential exacerbation of existing infections and the emergence of new infections due to immunosuppression (Sanskriti et al., 2021; Fishman, 2007; Borges et al., 2022). In our study, we found no statistically significant difference in the incidence of new infections between the CNI and non-CNI groups. Although the database did not provide direct evidence of whether CNIs exacerbated existing infections, the clear improvement in overall patient outcomes strongly suggests that the use of CNIs did not worsen pre-existing infections. This indicates a level of safety and reliability in using these drugs for septic patients.

The mechanism by which CNIs induce hypertension is linked to the overactivity of the renal sodium chloride cotransporter (NCC) and increased phosphorylation of N-methyl-D-aspartate (NMDA) receptors in the paraventricular nucleus (PVN) of the hypothalamus, leading to heightened sympathetic output (Hoorn et al., 2011; Zhou et al., 2022). Although the CNI group experienced an increase in hypertension, the mild elevation in blood pressure appears to have limited clinical implications for critically ill patients. Severe hypertension (SBP > 180 mmHg or DBP > 110 mmHg) can significantly increase the risk of acute cardiovascular and cerebrovascular events, but no significant differences in such life-threatening events were observed between the two groups. On the other hand, the blood pressure elevation caused by CNIs might also have a beneficial effect on improving septic shock.

CNIs have also shown beneficial effects on the cardiovascular system. The activation of calcineurin, a component in ventricular remodeling, suggests that tacrolimus may slow the progression of ventricular hypertrophy in mice, preventing the transition to heart failure (Sakata et al., 2000). Cyclosporine and tacrolimus have been found to inhibit endotoxin-mediated cardiac contractile dysfunction by reducing nitric oxide (NO) production and preserving mitochondrial function (Mandar et al., 2006). Additionally, a randomized controlled study demonstrated that cyclosporine pre-treatment in patients with acute myocardial infarction undergoing percutaneous coronary intervention (PCI) can reduce the opening of the mitochondrial permeability transition pore, thereby reducing myocardial damage during reperfusion and limiting the area of myocardial infarction (Christophe et al., 2008). These mechanisms may help protect organ function during sepsis, improving both short- and long-term outcomes for patients.

Hyperkalemia is another common adverse reaction attributed to CNIs, though its mechanism is not fully understood (Fleming et al., 1997; Tantisattamo et al., 2016). It is currently believed to be related to reduced aldosterone secretion or resistance, as well as the inhibition of inward rectifying potassium channels (Kir) in renal tubules (Berber et al., 2021; Kortenoeven, 2023). Some studies suggest that hormone replacement with fludrocortisone and the use of statin drugs can mitigate these inhibitory effects on potassium channels, offering a therapeutic effect for CNI-induced hyperkalemia (Ma, 2013; Unsal et al., 2021; Gheith et al., 2022). In our study, the CNI group showed a mild elevation in serum potassium compared to the control group, with no significant difference in the incidence of hyperkalemia. This may be due to the more stringent electrolyte monitoring typically conducted in ICU patients. However, given the myocardial toxicity associated with elevated potassium levels, clinicians should closely monitor electrolyte status in patients receiving CNIs.

Liver and kidney damage from CNIs also warrants attention. Reports on CNI-induced hepatotoxicity are relatively few, possibly due to the liver’s strong compensatory and regenerative abilities. This side effect mainly manifests as enzyme elevation and does not seem to significantly affect essential liver functions, thus having minimal impact on patient prognosis (El-Magd et al., 2022; Bingül et al., 2019). In our study, the CNI group showed an increased likelihood of liver injury (defined as ALT or AST levels exceeding five times the upper limit of normal) compared to the control group. Clinicians should be vigilant about this and may consider administering hepatoprotective drugs, particularly for patients with pre-existing chronic liver dysfunction. More attention is generally directed toward CNI-induced renal damage, primarily manifesting as chronic toxicity characterized by renal vascular injury, thrombotic microangiopathy, and interstitial fibrosis (Nagao et al., 2021; Bk and Betiuk, 1986; Nahman et al., 1987). Although this study did not find differences in the incidence of acute kidney injury (AKI) or the need for continuous renal replacement therapy (CRRT) between the two groups, the pre-matched cohort indicated that the CNI group had more baseline characteristics of chronic kidney disease (CKD). This renal toxicity could impact patient prognosis, especially in the long term. The use of statins and other renal protective drugs, such as glutathione, may help mitigate this renal damage (Huang et al., 2018; Mahmoud et al., 2022; Deger et al., 2021; Khanna et al., 2004).

This study has several limitations: 1) It is a retrospective cohort study based on electronic medical records from a single center, which may introduce residual confounding due to unmeasured covariates. 2) CNIs have potent anti-inflammatory and immunosuppressive effects, which could increase the risk of new infections or exacerbate existing ones in individual patients. Given the heterogeneity of sepsis, responses to CNI treatment may vary depending on the pathogen and disease state. Further research, including animal studies or larger observational trials, is necessary to better assess the safety and efficacy of CNIs, and to clarify critical parameters such as timing, dosage, and indications for their use. 3) The specific impact of CNIs on the progression of sepsis remains unclear. Immunological parameters, such as cytokine levels and leukocyte subtypes, were not available in the database. Future studies exploring the cellular and molecular mechanisms of CNIs in sepsis could provide deeper insights into the findings of this research.

## 5 Conclusion

In summary, the use of CNIs in septic patients significantly reduces both 28- and 365-day mortality and facilitates earlier liberation from invasive mechanical ventilation and vasoactive drugs. Aside from a mild elevation in liver enzymes, no significant increase in severe adverse reactions, such as new-onset infections, hyperkalemia, severe hypertension, or acute kidney injury (AKI), was observed. However, further research is essential to fully understand how CNIs improve outcomes in septic patients. Larger studies are needed to confirm their safety and efficacy, as well as to determine the optimal dosage, timing, and indications for their use.

## Data Availability

The original contributions presented in the study are included in the article/supplementary material, further inquiries can be directed to the corresponding author.

## Abbreviations

MIMIC-IV, Medical Information Mart for Intensive Care IV; CNIs, calcineurin inhibitors; AIDS, acquired immune deficiency syndrome; CNI, Calcineurin inhibitor; ICU, intensive care unit; MAP, mean arterial pressure; WBC, write blood cell; SOFA, Sequential Organ Failure Assessment; SAPSII, Simplified Acute Physiology Score; CCI, charlson comorbidity index; COPD, chronic obstructive pulmonary disease; CKD, chronic kidney disease; MV, mechanic ventilation; CRRT, continuous renal replacement therapy; GC, Glucocorticoid.
